# Supplementary material for: Theoretical Kinetic Study of Thermal Decomposition of 5-Methyl-2-ethylfuran
Source: Molecules. 2025 Apr 2;30(7):1595. doi: 10.3390/molecules30071595 (PMC11990546; doi:10.3390/molecules30071595)
Supplement: Supplementary file 1 [file molecules-30-01595-s001.zip › molecules-3529931-supplementary.pdf]

SUPPLEMENTARY MATERIALS TO

Theoretical Kinetic Study of Thermal Decomposition of 5-Methyl-2-ethylfuran

Wei He [a,b,c], Cheng Wang [b,c], Qichuan Zhang [b,c], Kaixuan Chen [b,c], Linghao Shen [a], Yan Li [b,c], Kang Shen [b,d,*]

[a]Eastern Michigan Joint College of Engineering, Beibu Gulf University, Qinzhou 535011, China

[b]Guangxi Key Laboratory of Ocean Engineering Equipment and Technology, Qinzhou 535011, China

[c]Key Laboratory of Beibu Gulf Offshore Engineering Equipment and Technology, Beibu Gulf University, Education Department of Guangxi Zhuang Autonomous Region, Qinzhou 535011, China

[d]College of Electrical Engineering, Guangxi University, Nanning 530004, China

Corresponding author:

Email: shenkang945@bbgu.edu.cn

Table S1 Rate constants for the unimolecular decomposition and intramolecular H-transfer decomposition reactions of 5-Methyl-2-ethylfuran, $k = AT^n\exp(-E_a/RT)$. Units are in cm^3, s and $cal \cdot mol^{-1}$.

**Selected Reactions**	*A*	*n*	E_a	Pressure (atm)
**5-MEF = h34d2o**				
	1.26E+19	-1.95	70355.17	0.01
	4.34E+18	-1.77	70398.40	0.039
	2.07E+19	-1.96	70800.44	0.1
	1.79E+15	-0.72	69336.42	1
	4.67E+14	-0.54	69122.44	10
	4.06E+14	-0.52	69100.26	100
**5-MEF = h45d3o**				
	9.96E+23	-3.58	71676.80	0.01
	1.48E+21	-2.65	71035.23	0.039
	1.33E+20	-2.30	70851.92	0.1
	1.21E+18	-1.61	70506.06	1
	4.57E+15	-0.84	69784.33	10
	4.23E+14	-0.53	69412.60	100
**5-MEF = 5mef2y+C_2H_5**				
	5.28E+36	-6.54	93157.30	0.01
	3.46E+30	-4.63	91038.34	0.039
	4.65E+26	-3.44	89661.61	0.1
	8.32E+19	-1.38	87172.25	1
	1.16E+18	-0.82	86469.35	10
	6.77E+17	-0.75	86380.51	100
**5-MEF = 5ef2ym+ H**				
	1.88E+28	-4.55	74689.01	0.01
	2.83E+22	-2.76	72643.09	0.039
	1.75E+19	-1.78	71474.87	0.1
	2.76E+15	-0.62	70057.01	1
	7.04E+14	-0.44	69832.90	10
	6.09E+14	-0.42	69809.06	100
**5-MEF = 5ef2y + CH_3**				
	1.33E+31	-4.90	93813.71	0.01
	2.68E+25	-3.15	91788.39	0.039
	1.70E+22	-2.17	90615.20	0.1
	1.23E+18	-0.91	89061.16	1
	2.32E+17	-0.69	88784.57	10
	1.93E+17	-0.67	88754.31	100
**5-MEF = 5e2mf3y + H**				

	9.25E+19	-1.80	91302.09	0.01
	1.64E+17	-0.97	90268.62	0.039
	2.32E+16	-0.71	89945.39	0.1
	6.02E+15	-0.53	89722.42	1
	5.20E+15	-0.51	89698.44	10
	5.13E+15	-0.51	89696.02	100
**5-MEF = 2e5mf3y + H**				
	5.86E+19	-1.74	91614.61	0.01
	1.37E+17	-0.94	90624.89	0.039
	2.18E+16	-0.70	90321.37	0.1
	6.22E+15	-0.54	90114.12	1
	5.44E+15	-0.52	90091.94	10
	5.37E+15	-0.52	90089.70	100
**5-MEF = 5mf2ym + CH_3**				
	3.20E+35	-6.53	69681.43	0.01
	1.15E+33	-5.71	69211.61	0.039
	2.55E+32	-5.47	69240.22	0.1
	1.92E+28	-4.13	68282.26	1
	5.41E+19	-1.51	65230.73	10
	5.17E+16	-0.59	64106.27	100
**5-MEF = 25mf2ye + H**				
	1.79E+26	-3.83	79753.44	0.01
	7.18E+20	-2.18	77814.94	0.039
	1.46E+18	-1.36	76820.01	0.1
	2.55E+15	-0.52	75782.89	1
	1.06E+15	-0.41	75638.26	10
	9.67E+14	-0.40	75623.19	100
**5-MEF = 15mf2ye + H**				
	1.06E+28	-4.47	72438.53	0.01
	1.13E+25	-3.52	71583.37	0.039
	2.32E+21	-2.39	70265.65	0.1
	1.16E+16	-0.78	68308.24	1
	1.11E+15	-0.47	67924.08	10
	8.55E+14	-0.43	67881.37	100

Table S2 Rate constant for the H-abstraction reaction of 5-Methyl-2-ethylfuran with H atoms, $k = AT^n\exp(-E_a/RT)$. Units are in cm^3, s and $cal\cdot mol^{-1}$.

**Selected Reactions**	*A*	*n*	E_a	Pressure (atm)
**5-MEF + H = 5ef2ym + H_2**				
	1.55E+05	2.50	3180.14	-
**5-MEF + H = 15mf2ye + H2**				

	1.49E+07	1.95	2766.22	-
**5-MEF + H = 25mf2ye + H_2**				
	3.74E+02	3.39	6581.49	-
**5-MEF + H = 2e5mf3y + H_2**				
	2.45E+07	2.08	18713.21	-
**5-MEF + H = 5e2mf3y + H_2**				
	3.47E+07	2.07	18592.74	-

Table S3 Rate constant for the H-abstraction reaction of 5-Methyl-2-ethylfuran with CH_3, $k = AT^n\exp(-E_a/RT)$. Units are in cm^3, s and cal·mol^{-1}.

**Selected Reactions**	*A*	*n*	E_a	Pressure (atm)
**5-MEF + CH_3 = 5ef2ym + CH_4**				
	6.03E-07	5.43	2767.77	-
**5-MEF + CH_3 = 15mf2ye + CH_4**				
	6.59E-05	4.84	2370.58	-
**5-MEF + CH_3 = 25mf2ye + CH_4**				
	8.15E-12	7.04	4558.15	-
**5-MEF + CH_3 = 2e5mf3y + CH_4**				
	4.16E-02	4.21	15487.69	-
**5-MEF + CH_3 = 5e2mf3y + CH_4**				
	4.23E-02	4.18	15570.49	-

Table S4 Rate constant for the addition of 5-Methyl-2-ethylfuran to H atoms., $k = AT^n\exp(-E_a/RT)$. Units are in cm^3, s and cal·mol^{-1}.

**Selected Reactions**	*A*	*n*	E_a	Pressure (atm)
**5-MEF+H=$CH_3CCHCH_2COC_2H_5$**				
	1.48E-02	-2.83	37122.60	0.01
	1.11E-06	-1.56	35666.45	0.039
	1.91E-07	-1.32	35511.76	0.1
	5.83E-06	-1.72	36902.08	1
	1.78E-04	-2.09	39024.40	10
	1.75E-08	-0.83	39488.81	100
**5-MEF + H = MF2+ C_2H_3**				
	3.83E-01	-3.10	10890.32	0.01
	1.82E-02	-2.68	11026.31	0.039

	5.61E-04	-2.22	10895.39	0.1
	3.36E-09	-0.65	9954.74	1
	2.95E-14	0.83	8832.95	10
	3.98E-10	-0.45	11687.61	100
**5-MEF+ H = $CH_3COCHCHCHC_2H_5$**				
	2.85E+10	-7.24	7529.54	0.01
	2.72E+09	-6.79	7865.98	0.039
	2.99E+09	-6.72	8405.56	0.1
	5.27E+05	-5.33	8922.90	1
	3.65E+02	-4.17	9850.50	10
	1.03E-01	-2.98	10693.75	100
**5-MEF+ H = $CH_3COCH_2CHCC_2H_5$**				
	1.12E+15	-8.69	19608.31	0.01
	3.14E+11	-7.34	19841.91	0.039
	1.64E+06	-5.49	19304.55	0.1
	4.68E+00	-3.47	19802.47	1
	5.17E-07	-1.14	19661.81	10
	2.39E-04	-1.91	22716.12	100
**5-MEF + H= $CH_3CHCHCHCOC_2H_5$**				
	3.72E+01	-4.14	6327.20	0.01
	2.59E-01	-3.39	6230.54	0.039
	1.12E-01	-3.22	6598.17	0.1
	1.01E+00	-3.37	8446.10	1
	8.92E-03	-2.65	9394.10	10
	3.77E-06	-1.57	10128.84	100
	3.72E+01	-4.14	6327.20	100

Table S5 The chemical structures of important species discussed in this work. Names in italics refer to the name assigned in the text.

Mechanism Name	Structure
**5MEF**	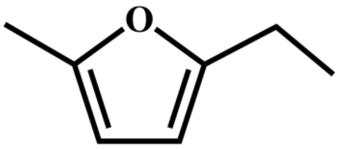
**5ef2y**	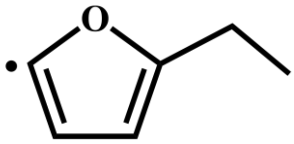

5mf2ym

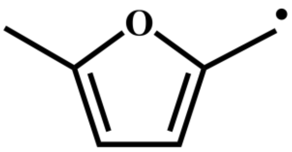

5mef2y

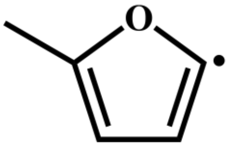

5ef2ym

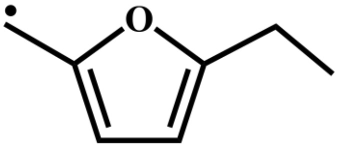

15mf2ye

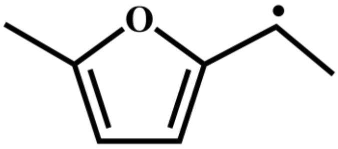

25mf2ye

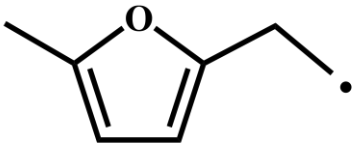

2e5mf3y

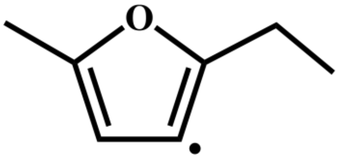

5e2mf3y

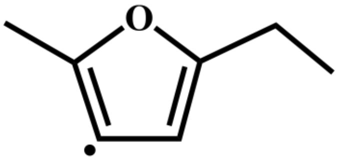

Table S6 Quantum chemistry calculations include the geometry, frequency and energy of stable structures and transition states

	**5MEF**			**Frequencies**		
C	2.14360200	-0.60639000	0.00034000	57.08	135.23	169.98
C	2.17467900	0.75687700	0.00016600	177.44	238.06	293.15
C	0.81339700	1.17319600	-0.00018100	306.53	448.96	628.38
C	0.05187000	0.03230200	-0.00008500	630.22	640.1	686.78
O	0.87346400	-1.06145400	0.00026600	790.43	808.31	847.26
H	3.05437400	1.38014000	0.00023100	948.6	969.33	995.98
H	0.44440100	2.18639100	-0.00041800	1024.08	1043.31	1065.15
C	-1.40030000	-0.21132300	-0.00021700	1083.99	1114.03	1199.44

C	-2.28795200	1.02188600	0.00023400	1234.48	1236.56	1283.1
H	-2.09865000	1.63893000	0.88325900	1350.46	1401.31	1416.83
H	-2.09869100	1.63954300	-0.88237100	1422.67	1474.18	1488.79
H	-3.32773500	0.69902100	0.00015000	1494.6	1499.82	1510.72
H	-1.63904524	-0.79128425	-0.86713433	1602.74	1654.33	3008.72
H	-1.63909094	-0.79195106	0.86624128	3021.43	3027.52	3033.13
C	3.24340335	-1.68436814	0.00068590	3066.23	3096.19	3101.17
H	4.10966282	-1.30994394	0.50496862	3115.93	3236.52	3251.78
H	2.88657569	-2.55779183	0.50536903			
H	3.49811925	-1.93435344	-1.00803961			

	**TS1**				**Frequencies**	
C	-1.61121200	0.02454200	0.00015900	-994.49	47.19	122.45
C	-1.16086500	1.30619700	-0.00010700	164.79	177.91	215.3
C	0.27422300	1.24022500	-0.00059600	275.8	303.72	367.84
C	0.60353900	-0.07907800	-0.00039400	439.8	530.15	628.78
O	-0.54174400	-0.83410000	-0.00026000	634.73	658.69	699.58
H	-1.77276200	2.19474200	-0.00002400	788.64	807.71	856.52
H	0.96145300	2.07091600	-0.00075500	950.9	969.13	999.43
C	1.88976500	-0.83585200	-0.00086300	1024.26	1043.49	1061.84
C	3.12531300	0.06744300	0.00115900	1084.06	1112.48	1203.62
H	3.14644800	0.71117300	-0.88196200	1235.45	1252.04	1282.24
H	3.14538600	0.70865500	0.88613400	1295.92	1351.26	1370.36
H	4.03805600	-0.53227700	0.00081500	1409.63	1419.52	1434.09
H	1.91177100	-1.49916400	0.87249900	1469.44	1486.28	1500.57
H	1.91283200	-1.49650500	-0.87624100	1510.46	1586.62	1635.09
C	-2.96306400	-0.59384000	0.00058200	3011.02	3030.57	3034.23
H	-3.72770500	0.18429800	0.00156500	3079.21	3097.42	3103.24
H	-3.16320097	-1.42584420	-1.16774905	3151.32	3239.17	3254.01
H	-3.11336700	-1.22407900	0.88301500			

	**TS2**				**Frequencies**	
C	-1.61121200	0.02454200	0.00015900	-647.75	64.56	126
C	-1.16086500	1.30619700	-0.00010700	153.62	183.23	223.27
C	0.27422300	1.24022500	-0.00059600	247.15	273.91	313.48
C	0.60353900	-0.07907800	-0.00039400	372.52	454.78	630.13
O	-0.54174400	-0.83410000	-0.00026000	637.38	661.82	693.59
H	-1.77276200	2.19474200	-0.00002400	799.03	841.61	857.5
H	0.96145300	2.07091600	-0.00075500	949.99	973.5	1000.56
C	1.88976500	-0.83585200	-0.00086300	1033	1044.83	1063.54
C	3.12531300	0.06744300	0.00115900	1085.45	1102.79	1207.05
H	3.14644800	0.71117300	-0.88196200	1234.81	1238.4	1260.05
H	3.14538600	0.70865500	0.88613400	1357.76	1382.7	1404.69
H	4.03805600	-0.53227700	0.00081500	1416.04	1424.4	1473.28

H	1.91177100	-1.49916400	0.87249900	1492.35	1496.98	1504.18
H	1.91924901	-1.68029210	-1.11976247	1548.95	1635.69	1699.63
C	-2.96306400	-0.59384000	0.00058200	3023.51	3031.97	3061.32
H	-3.72770500	0.18429800	0.00156500	3069.38	3093.71	3104.14
H	-3.11435500	-1.22278300	-0.88260300	3118.43	3239.21	3254.08
H	-3.11336700	-1.22407900	0.88301500			
H	1.94399110	-2.38892075	-2.05870913			

	**TS3**				**Frequencies**	
C	-1.82468288	0.08028469	0.00001187	-1171.86	37.55	105.1
C	-1.42519388	1.37878269	-0.00000813	135.84	157.57	187.83
C	0.01130112	1.36988069	-0.00005213	277.35	285.71	325.04
C	0.39094112	0.06485369	-0.00005613	446.08	572.95	627.24
O	-0.72218788	-0.73594531	-0.00001613	638.65	650.06	699.02
H	-2.07149688	2.24252169	0.00000887	793.29	813.55	850.95
H	0.66720012	2.22573469	-0.00007813	956.18	971.3	993.2
C	1.70821912	-0.64669531	-0.00011213	1015.83	1043.3	1062.99
C	2.89216612	0.29162869	0.00011287	1065.23	1106.06	1189.94
H	3.03431912	0.87696469	-0.90681113	1191.17	1193.09	1232.33
H	3.03413612	0.87674469	0.90720787	1235.22	1287.2	1327.48
H	4.14061600	-0.60025100	0.00012400	1395.83	1420.42	1468.81
H	1.75391012	-1.30541031	0.87582287	1474.36	1486.04	1494.27
C	-3.15118088	-0.59071431	0.00005887	1598.77	1653.42	1742.75
H	-3.94551288	0.15700869	0.00006687	3007.7	3022.58	3032.29
H	-3.27691988	-1.22557831	-0.88277013	3067.95	3085.34	3116.79
H	-3.27687388	-1.22554831	0.88291587	3167.01	3237.57	3252.01
H	1.75401412	-1.30511931	-0.87626113			
H	5.02967636	-1.29626715	0.00010672			

	**TS4**				**Frequencies**	
C	-1.61121200	0.02454200	0.00015900	-641.81	53.47	126.8
C	-1.16086500	1.30619700	-0.00010700	140.45	172.21	173.67
C	0.27422300	1.24022500	-0.00059600	209.8	242.2	284.54
C	0.60353900	-0.07907800	-0.00039400	304.54	449.12	578.28
O	-0.54174400	-0.83410000	-0.00026000	630.23	633.58	681.08
H	-1.77276200	2.19474200	-0.00002400	773.25	797.89	802.54
H	1.17186101	2.32524721	-0.00080368	882.62	925.32	961.09
C	1.88976500	-0.83585200	-0.00086300	997.89	1025.07	1060.56
C	3.12531300	0.06744300	0.00115900	1069.24	1113.76	1129.71
H	3.11606831	0.68637298	-0.87161830	1167.55	1219.43	1283.13
H	3.11502632	0.68388697	0.87568231	1327.67	1383.7	1416.64

H	1.92030259	-1.45991299	0.86776542	1419.43	1473.26	1488.1
C	-2.96306400	-0.59384000	0.00058200	1490.71	1500.05	1510.3
H	-3.72770500	0.18429800	0.00156500	1597.22	1621.56	2902.32
H	-3.11435500	-1.22278300	-0.88260300	3010.46	3022.75	3029.97
H	-3.11336700	-1.22407900	0.88301500	3037.27	3069.19	3103.15
H	1.92122035	-1.45727536	-0.87134767	3103.75	3116.97	3248.03
H	4.00862070	-0.53643397	0.00082658			
H	1.88215645	3.18381862	-0.00096802			
		**TS5**			**Frequencies**	
C	-1.61121200	0.02454200	0.00015900	-625.87	51.85	129.51
C	-1.16086500	1.30619700	-0.00010700	133.35	159.38	180.45
C	0.27422300	1.24022500	-0.00059600	184.9	233.33	284.98
C	0.60353900	-0.07907800	-0.00039400	309.6	453.21	581.05
O	-0.54174400	-0.83410000	-0.00026000	623.67	631.65	678.75
H	-1.96477474	2.47356664	0.00000205	764.05	783.66	817.54
C	1.88976500	-0.83585200	-0.00086300	886.94	936.54	950.78
C	3.12531300	0.06744300	0.00115900	983.67	1024.59	1063.7
H	3.11606831	0.68637298	-0.87161830	1082.78	1110.78	1136.95
H	3.11502632	0.68388697	0.87568231	1186.18	1206.92	1281.93
H	1.92030259	-1.45991299	0.86776542	1314.32	1385.5	1417.61
C	-2.96306400	-0.59384000	0.00058200	1420.39	1475.18	1485.84
H	-3.72770500	0.18429800	0.00156500	1490.58	1500.96	1510.09
H	-3.11435500	-1.22278300	-0.88260300	1590.5	1631.08	2914.66
H	-3.11336700	-1.22407900	0.88301500	3009.93	3024.07	3029.91
H	1.92122035	-1.45727536	-0.87134767	3033.86	3069.67	3096.93
H	4.00862070	-0.53643397	0.00082658	3102.85	3122.33	3256.65
H	0.95206088	2.06813539	-0.00103395			
H	-2.62785565	3.43643658	0.00009199			
		**TS6**			**Frequencies**	
C	-1.61121200	0.02454200	0.00015900	-1439.36	36.55	45.95
C	-1.16086500	1.30619700	-0.00010700	51.88	77.33	171.01
C	0.27422300	1.24022500	-0.00059600	177.54	244.75	296.45
C	0.60353900	-0.07907800	-0.00039400	304.2	346.31	440.39
O	-0.54174400	-0.83410000	-0.00026000	467.27	553.41	627.1
H	-1.77276200	2.19474200	-0.00002400	636.05	674.77	678.95
H	0.96145300	2.07091600	-0.00075500	712.71	786.91	804.27
C	1.88976500	-0.83585200	-0.00086300	851.58	951.4	969.55
C	3.12531300	0.06744300	0.00115900	1004.34	1025.45	1046.28
H	3.14644800	0.71117300	-0.88196200	1069.32	1083.79	1107.46
H	3.14538600	0.70865500	0.88613400	1111.98	1204.48	1236.92
H	4.03805600	-0.53227700	0.00081500	1258.39	1281.29	1354.4
H	1.91177100	-1.49916400	0.87249900	1394.91	1403.03	1413.73

H	1.91283200	-1.49650500	-0.87624100	1421.86	1431.68	1436.85
C	-2.96306400	-0.59384000	0.00058200	1450.67	1486.54	1499.89
H	-3.72770500	0.18429800	0.00156500	1510.61	1571.1	1629.59
H	-3.16320097	-1.42584420	-1.16774905	3007.8	3026.59	3033.05
H	-3.11336700	-1.22407900	0.88301500	3063.26	3078.14	3095.79
C	-3.47734920	-2.13795933	-2.26809798	3101.87	3150.88	3206.2
H	-3.12069477	-3.14676933	-2.26809798	3207.56	3237.58	3251.69
H	-3.12067636	-1.63356114	-3.14174949			
H	-4.54734920	-2.13794615	-2.26809798			
		**TS7**			**Frequencies**	
C	-1.61121200	0.02454200	0.00015900	-1327.99	42.7	46.91
C	-1.16086500	1.30619700	-0.00010700	69.52	129.23	135.76
C	0.27422300	1.24022500	-0.00059600	170.85	193.35	225.61
C	0.60353900	-0.07907800	-0.00039400	297.15	310.91	443.87
O	-0.54174400	-0.83410000	-0.00026000	456.11	517.37	556.61
H	-1.77276200	2.19474200	-0.00002400	630.84	643.31	682.87
H	0.96145300	2.07091600	-0.00075500	699.07	793.48	846.67
C	1.88976500	-0.83585200	-0.00086300	887.95	948.79	972.93
C	3.12531300	0.06744300	0.00115900	1002.96	1035.8	1048.24
H	3.14644800	0.71117300	-0.88196200	1063.55	1087.05	1102.9
H	3.14538600	0.70865500	0.88613400	1113.72	1206.4	1238.09
H	4.03805600	-0.53227700	0.00081500	1243.8	1320.4	1372.86
H	1.91177100	-1.49916400	0.87249900	1404.13	1414.52	1417.43
H	1.91924901	-1.68029210	-1.11976247	1423.78	1431.83	1444.8
C	-2.96306400	-0.59384000	0.00058200	1473.13	1492.81	1495.87
H	-3.72770500	0.18429800	0.00156500	1502.51	1572.81	1637.87
H	-3.11435500	-1.22278300	-0.88260300	3021.08	3023.08	3065.13
H	-3.11336700	-1.22407900	0.88301500	3065.65	3065.73	3081.66
C	1.71787788	-2.41619434	-2.21940429	3097.55	3117	3208.65
H	2.07453231	-3.42500434	-2.21940429	3210.63	3237.05	3251.95
H	2.07455072	-1.91179615	-3.09305579			
H	0.64787788	-2.41618115	-2.21940429			
		**TS8**			**Frequencies**	
C	2.32142100	-0.14475200	-0.00006700	-1639.51	25	31.68
C	2.09243700	1.19431900	-0.00009200	44.8	68.19	124.83
C	0.66669900	1.37039400	0.00006300	175.14	185.35	275.17
C	0.12107700	0.12540500	0.00017100	281.68	367.11	416.47
O	1.12318700	-0.81217000	0.00009200	502.99	557.46	626.82
H	2.84491100	1.96741700	-0.00021200	638.87	661.48	683.83
H	0.12588300	2.30310900	0.00008700	710.66	796.09	826.33
C	-1.27683000	-0.40863300	0.00039300	852.52	962.88	971.91
C	-2.34040100	0.67036700	-0.00007200	1009.69	1040.54	1055.04

H	-2.36540200	1.28330000	0.90133000
H	-2.36507900	1.28283100	-0.90180200
H	-3.52401700	0.06234500	-0.00017600
H	-1.40372200	-1.05853800	-0.87471400
C	3.55070600	-0.98090400	-0.00017100
H	4.43479400	-0.34170900	-0.00037400
H	3.59409000	-1.62678100	0.88268000
H	3.59381300	-1.62696100	-0.88290400
H	-1.40376300	-1.05779800	0.87604600
C	-4.73126200	-0.58372100	-0.00023300
H	-4.73464200	-1.17970300	0.90953600
H	-5.47042600	0.21399400	0.00028100
H	-4.73501600	-1.17898900	-0.91046500

1064.93	1081.94	1118.73
1153.62	1192.28	1229.41
1235.08	1285.41	1332.43
1381.64	1384.83	1396.01
1419.87	1447.69	1449.2
1468.8	1473.37	1484.57
1494.37	1599.08	1653.72
3001.09	3021.4	3023.66
3055.65	3066.21	3068.02
3115.75	3141.41	3185.58
3186.59	3237.55	3251.9

TS9

C	-1.61121200	0.02454200	0.00015900
C	-1.16086500	1.30619700	-0.00010700
C	0.27422300	1.24022500	-0.00059600
C	0.60353900	-0.07907800	-0.00039400
O	-0.54174400	-0.83410000	-0.00026000
H	-1.77276200	2.19474200	-0.00002400
H	1.11347183	2.25466915	-0.00079017
C	1.88976500	-0.83585200	-0.00086300
C	3.12531300	0.06744300	0.00115900
H	3.11606831	0.68637298	-0.87161830
H	3.11502632	0.68388697	0.87568231
H	1.92030259	-1.45991299	0.86776542
C	-2.96306400	-0.59384000	0.00058200
H	-3.72770500	0.18429800	0.00156500
H	-3.11435500	-1.22278300	-0.88260300
H	-3.11336700	-1.22407900	0.88301500
H	1.92122035	-1.45727536	-0.87134767
H	4.00862070	-0.53643397	0.00082658
C	1.94054563	3.25439668	-0.00098153
H	2.84104888	2.97221565	-0.50533036
H	1.49474704	4.08601004	-0.50554356
H	2.16789796	3.52940249	1.00777152

Frequencies

-1349.83	20.51	41.38
75.01	85.26	124.69
169.27	173.7	240.9
287.32	299.97	387.67
459.23	466.91	513.9
607.42	635.29	640.87
705.49	800.35	804.08
956.85	966.59	1003.82
1041.49	1061.49	1075.1
1112.37	1127.88	1167.55
1217.22	1233.69	1265
1283.06	1319.78	1358.66
1389.24	1415.37	1417.92
1452.3	1461.67	1473.08
1488.44	1492.11	1499.24
1509.96	1597.54	1628.89
3008.01	3021.03	3026.83
3036.37	3053.66	3066.31
3101.34	3102.3	3114.77
3173.26	3176.18	3237.63

TS10

C	-1.59908870	0.00693756	0.00015736
C	-1.14874170	1.28859256	-0.00010864
C	0.28634630	1.22262056	-0.00059764
C	0.61566230	-0.09668244	-0.00039564
O	-0.52962070	-0.85170444	-0.00026164

Frequencies

-1316.25	28.8	57.02
63.83	68.49	125.19
174.21	176.07	237.24
287.68	304.77	399.08
453.57	454.16	514.21

H	-1.91062398	2.39493350	-0.00000529	610.17	626.61	632.71
C	1.90188830	-0.85345644	-0.00086464	712.65	788.04	820.82
C	3.13743630	0.04983856	0.00115736	947.58	962.82	1007.06
H	3.12819161	0.66876854	-0.87161994	1039.76	1061.92	1083.59
H	3.12714962	0.66628253	0.87568067	1111.49	1131.91	1198.43
H	1.93242589	-1.47751743	0.86776378	1208.85	1219.31	1265.01
C	-2.95094070	-0.61144444	0.00058036	1281.71	1316.46	1352.76
H	-3.71558170	0.16669356	0.00156336	1390.05	1416.39	1420.92
H	-3.10223170	-1.24038744	-0.88260464	1453.04	1462.2	1474.17
H	-3.10124370	-1.24168344	0.88301336	1486.56	1492.18	1501.67
H	1.93334365	-1.47487980	-0.87134931	1510.83	1593.22	1634.56
H	4.02074400	-0.55403841	0.00082494	3008.3	3021.06	3027.68
H	0.96418418	2.05053095	-0.00103559	3033.37	3052.01	3065.44
C	-2.63792295	3.45105546	0.00009336	3096.25	3101.66	3118.58
H	-2.12067570	4.24036366	-0.50423576	3171.84	3173.71	3246.64
H	-3.55975297	3.24934233	-0.50432833			
H	-2.84021434	3.74471165	1.00892646			
		**TS11**			**Frequencies**	
C	-1.61461282	0.02902061	-0.48124396	-663.63	58.8	134.52
C	-1.16426582	1.31067561	-0.48150996	158.78	182.46	235.35
C	0.27082218	1.24470361	-0.48199896	279.15	304.44	353.36
C	0.60013818	-0.07459939	-0.48179696	399.92	451.5	623.75
O	-0.54514482	-0.82962139	-0.48166296	631.05	655.17	690.38
H	-1.77616282	2.19922061	-0.48142696	798.99	818.62	941.87
H	0.22462851	1.18803352	0.83807795	949.72	960.35	995.79
C	1.88636418	-0.83137339	-0.48226596	1016.46	1039.24	1066.24
C	3.12191218	0.07192161	-0.48024396	1083.1	1106.99	1207.92
H	3.14304718	0.71565161	-1.36336496	1222.31	1230.62	1283.08
H	3.14198518	0.71313361	0.40473104	1339.06	1397.7	1417.97
H	4.03465518	-0.52779839	-0.48058796	1423.01	1473.66	1480.69
H	1.90837018	-1.49468539	0.39109604	1493.1	1502.49	1512.11
H	1.90943118	-1.49202639	-1.35764396	1560.95	1652.3	2993.39
C	-2.96646482	-0.58936139	-0.48082096	3025.21	3034.58	3035.9
H	-3.73110582	0.18877661	-0.47983796	3071.91	3099.01	3103.53
H	-3.11775582	-1.21830439	-1.36400596	3119.55	3242.78	3253.61
H	-3.11676782	-1.21960039	0.40161204			
H	0.94866013	2.07261403	-0.48220145			
		**TS12**			**Frequencies**	
C	1.76308165	0.11871208	-0.00003331	-627.04	49.51	92.28
C	1.22560700	1.27520000	-0.00000400	136.11	189.55	203.84
C	-0.28342300	1.33590300	0.00003000	224.98	261.04	366.21
C	-0.61295100	-0.12621400	0.00004600	377.35	490.92	562.82

O	0.40921535	-1.00381708	-0.00011469	666.79	741.33	787.13
H	1.88198100	2.13210400	0.00002500	822.93	879.11	943.42
C	-1.89133700	-0.87989000	0.00009800	968.35	987.62	1028.77
C	-3.12926500	0.02426900	-0.00006100	1055.22	1056.8	1065.27
H	-3.15309600	0.66858400	0.88283700	1136.9	1192.47	1204.64
H	-3.15399200	0.66703300	-0.88406300	1257.84	1321.57	1335.02
H	-4.04230800	-0.57524200	0.00094100	1392.75	1411.47	1443.36
H	-1.92874300	-1.55055800	-0.87324400	1459.49	1469.21	1473.17
H	-1.92881200	-1.55037700	0.87357600	1477.07	1500.85	1506.18
C	3.10733865	-0.51667592	0.00003769	1784.04	2951.52	2976.58
H	3.88924065	0.24336808	-0.00031231	2992.27	3026.82	3035.72
H	3.23413665	-1.15193192	0.88194369	3047.42	3070.45	3074.85
H	3.23391765	-1.15253192	-0.88146331	3093.86	3096.35	3128.85
H	-0.68440100	1.87549300	-0.87676000			
H	-0.68438300	1.87549400	0.87681600			
		**TS13**			**Frequencies**	
C	-1.62342110	0.01821467	-0.22952973	-436.69	71.74	128.71
C	-1.17307410	1.29986967	-0.22979573	162.06	204.14	244.8
C	0.26201390	1.23389767	-0.23028473	303.6	312.52	365.16
C	0.59132990	-0.08540533	-0.23008273	420.54	452.01	611.67
O	-0.55395310	-0.84042733	-0.22994873	633.7	653.96	690.3
H	-1.78497110	2.18841467	-0.22971273	788.31	806.52	857.29
H	0.94924390	2.06458867	-0.23044373	943.91	971.92	995.2
C	1.87755590	-0.84217933	-0.23055173	1023.23	1042.1	1062.31
C	3.11310390	0.06111567	-0.22852973	1082.86	1106.84	1195.38
H	3.13423890	0.70484567	-1.11165073	1234.46	1240.68	1285.94
H	3.13317690	0.70232767	0.65644527	1357.07	1401.63	1415.12
H	4.02584690	-0.53860433	-0.22887373	1421.5	1473.17	1487.16
H	1.89956190	-1.50549133	0.64281027	1491.94	1500.96	1510.86
H	1.90062290	-1.50283233	-1.10592973	1554.56	1626	3007.22
C	-2.97527310	-0.60016733	-0.22910673	3024.28	3032.79	3057.28
H	-3.73991410	0.17797067	-0.22812373	3070.57	3096.57	3103.52
H	-3.12656410	-1.22911033	-1.11229173	3119.34	3240.01	3256.11
H	-3.12557610	-1.23040633	0.65332627			
H	0.66031319	-0.04965497	1.06769353			
		**TS14**			**Frequencies**	
C	-1.66798046	0.11105543	0.02288583	-462.18	51.18	92.5
C	-1.15713146	1.39814643	0.02182283	112.92	141.83	164.85
C	0.14207654	1.36464943	0.50294283	199.89	252.64	340.78
C	0.46804854	-0.05663857	0.86379283	525.42	568.69	619.84
O	-0.74702446	-0.78544357	0.48901283	657.94	742.98	779.82
H	-1.69952446	2.27158343	-0.31085017	832.69	835.95	851.13

H	0.80887154	2.19930643	0.65210783	891.56	933.34	984.95
C	2.02268646	-0.91442843	0.01206517	1016.63	1037.77	1058.42
C	2.88031746	-0.01358343	-0.88236683	1063.1	1080.57	1134.83
H	2.29066046	0.42832557	-1.68860483	1215.29	1245.52	1253.62
H	3.33607546	0.80473657	-0.31773483	1400.04	1403.68	1418.39
H	3.69071346	-0.59266143	-1.33330983	1442.84	1466.52	1471.62
H	2.65211646	-1.35732443	0.78994517	1485.92	1489.97	1494.25
H	1.62866546	-1.74766343	-0.57529583	1603.12	2966.59	3017.97
C	-2.97648846	-0.44389157	-0.39862617	3035.45	3061.08	3076.12
H	-3.63529346	0.35233543	-0.74824017	3106.3	3116.09	3194.94
H	-2.85400846	-1.17413357	-1.20764917	3232.2	3238.7	3254.66
H	-3.46983946	-0.96561357	0.42973783			
H	0.55756854	-0.17888657	1.95384283			
		**TS15**			**Frequencies**	
C	-1.50491700	0.02107000	-0.06646600	-469.8	31.88	112.88
C	-0.99419000	1.30820700	-0.06751100	127.72	173.7	208.65
C	0.30500300	1.27485700	0.41350700	269.66	347.12	380.35
C	0.79854983	-0.04603650	0.82625689	517.5	591.47	616.13
O	-0.75139283	-0.97588550	0.34788811	672.35	723.04	768.67
H	-1.53668000	2.18155800	-0.40025900	829.35	873.88	955.19
H	0.97155100	2.10958300	0.56332600	976.79	1017.72	1023.36
C	2.02731083	-0.72391850	0.15329489	1045.94	1072.5	1085.4
C	2.88467083	0.17679650	-0.74153111	1119.18	1191.14	1253.98
H	2.29486483	0.61822250	-1.54792511	1286.63	1321.95	1361.99
H	3.34022183	0.99547050	-0.17724811	1398.32	1403.48	1413.56
H	3.69521383	-0.40224050	-1.19226311	1457.27	1468.15	1472.92
H	2.65687183	-1.16637350	0.93132289	1494.63	1496.9	1500.05
H	1.63344283	-1.55746050	-0.43373811	1508.36	2979.52	3026.63
C	-2.81343800	-0.53395300	-0.48783700	3034.14	3058.97	3079.45
H	-3.47193800	0.26209800	-0.83842500	3097.61	3105.25	3106.92
H	-2.69085800	-1.26500300	-1.29610500	3113.39	3146.99	3204.53
H	-3.30721100	-1.05475500	0.34086400			
H	0.88815483	-0.16810050	1.91633389			
		**TS16**			**Frequencies**	
C	-1.59987157	0.05155564	-0.22857232	-673.2	58.06	102.92
C	-1.14952457	1.33321064	-0.22883832	165.54	177.37	227.23
C	0.28556343	1.26723864	-0.22932732	272.51	303.86	354.36
C	0.61487943	-0.05206436	-0.22912532	391.25	451.9	627.61
O	-0.53040357	-0.80708636	-0.22899132	627.98	655.97	689.7
H	-1.74538956	2.21655035	-0.13108417	794.22	828.14	942.1
H	0.97279343	2.09792964	-0.22948632	944.78	966.79	988.12
C	1.90110543	-0.80883836	-0.22959432	1018.77	1035.23	1057.22

C	3.13665343	0.09445664	-0.22757232	1085.04	1114.96	1184.8
H	3.15778843	0.73818664	-1.11069332	1229.03	1242.94	1283.5
H	3.15672643	0.73566864	0.65740268	1340.03	1397.63	1415.67
H	4.04939643	-0.50526336	-0.22791632	1420.89	1471.5	1485.75
H	1.92311143	-1.47215036	0.64376768	1488.33	1501.09	1511.41
H	1.92417243	-1.46949136	-1.10497232	1572.22	1643.26	3012.19
C	-2.95172357	-0.56682636	-0.22814932	3015.75	3032.14	3034.71
H	-3.71636457	0.21131164	-0.22716632	3066.34	3098.24	3103.44
H	-3.10301457	-1.19576936	-1.11133432	3120.05	3238.09	3257.76
H	-3.10202657	-1.19706536	0.65428368			
H	-1.21348538	1.18085182	1.06122220			
		**TS17**			**Frequencies**	
C	-1.61150400	-0.05390500	0.14766300	-610.58	43.01	96.02
C	-1.18121100	1.37652400	0.00432600	114.47	139.35	179.84
C	0.32177200	1.21391500	0.00270900	252.66	335.93	364.19
C	0.79504645	0.03415068	0.01412200	380.03	482.68	570.65
O	-0.67748645	-1.01394268	0.05896800	644.66	730.2	800.18
H	-1.54446500	2.01158500	0.82753400	820.27	872.65	919.32
H	1.03300300	2.02431600	-0.02298900	932.14	987.51	1016.83
C	2.08592045	-0.71393632	0.00849000	1025.43	1060.75	1102.5
C	3.32665445	0.17712568	-0.05897800	1132.47	1189.21	1204.31
H	3.31139645	0.80914568	-0.95051600	1278.23	1310.93	1334.99
H	3.39345645	0.82910168	0.81574800	1386.89	1403.28	1425.11
H	4.23334945	-0.43078332	-0.09310800	1469.14	1470.73	1475.64
H	2.11889045	-1.34367432	0.90574300	1487.21	1496.06	1505.71
H	2.07139745	-1.40987132	-0.83896400	1774.59	2965.24	2990.28
C	-2.93361400	-0.67661200	-0.08737100	3007.77	3027.91	3030.58
H	-2.98059700	-1.19930600	-1.05620000	3036.78	3059.3	3092.86
H	-3.18167200	-1.41772700	0.68289300	3101.29	3124.57	3135.4
H	-3.71368100	0.08723700	-0.08030400			
H	-1.55083200	1.85050700	-0.92618800			
		**TS18**			**Frequencies**	
C	-1.60130323	0.01835667	-0.20550956	-464.99	53.4	156.12
C	-1.15095623	1.30001167	-0.20577556	175.99	189.33	246.85
C	0.28413177	1.23403967	-0.20626456	295.06	310.2	350.41
C	0.61344777	-0.08526333	-0.20606256	424.8	454.75	617.27
O	-0.53183523	-0.84028533	-0.20592856	633.61	642.94	690.82
H	-1.76285323	2.18855667	-0.20569256	787.47	807.14	857.66
H	0.97136177	2.06473067	-0.20642356	948.48	963.02	993.04
C	1.89967377	-0.84203733	-0.20653156	1022.77	1042.52	1058.76
C	3.13522177	0.06125767	-0.20450956	1083.85	1112.15	1204.12
H	3.15635677	0.70498767	-1.08763056	1234.2	1235.33	1282.71

H	3.15529477	0.70246967	0.68046544	1354.81	1404.38	1415.46
H	4.04796477	-0.53846233	-0.20485356	1421.1	1476.55	1485.38
H	1.92167977	-1.50534933	0.66683044	1492.64	1500.64	1510.29
H	1.92274077	-1.50269033	-1.08190956	1558.91	1618.45	3011.62
C	-2.95315523	-0.60002533	-0.20508656	3024.51	3031.3	3034.32
H	-3.71779623	0.17811267	-0.20410356	3090.25	3097.59	3103.27
H	-3.10444623	-1.22896833	-1.08827156	3125.41	3240.64	3255.33
H	-3.10345823	-1.23026433	0.67734644			
H	-1.66267991	0.05666966	1.06843746			

	**TS19**				**Frequencies**	
C	-1.77997284	0.04815526	0.52832595	-479.63	54.44	100.66
C	-1.20182700	1.32267500	0.09142800	131.42	206.92	226.24
C	0.12675800	1.28182000	-0.29409700	253.91	343.09	382.61
C	0.59585600	-0.01813700	-0.16414200	457.84	569.27	651.37
O	-0.21591216	-0.96464726	0.26399805	701.85	709.64	797.56
H	-1.85901300	2.17845300	0.11824500	832.84	883.66	934.52
H	0.71859200	2.11358500	-0.64900500	994.91	997.82	1038.29
C	1.92114800	-0.64179300	-0.45202600	1076.09	1082.94	1089.38
C	3.08862500	-0.01325700	0.32846500	1148.03	1185.84	1240.72
H	3.19746500	1.04846500	0.09313700	1277.16	1329.09	1344.63
H	2.93016900	-0.10189500	1.40586700	1394.8	1404.19	1416.36
H	4.03080900	-0.50913600	0.07842700	1458.12	1477.52	1478.99
H	1.84136400	-1.70516100	-0.21308100	1486.82	1497.05	1509.5
H	2.13539600	-0.58006100	-1.52713800	1511.56	3009.32	3018.89
C	-2.92242884	-0.57979374	-0.26926205	3031.54	3066.08	3080.88
H	-3.84934984	-0.02699474	-0.09182705	3093.78	3100.2	3113.79
H	-2.69894684	-0.55090374	-1.33786905	3121.09	3146.92	3204.57
H	-3.07466384	-1.61858774	0.03353995			
H	-2.01389084	-0.02175674	1.60223295			

	**TS20**				**Frequencies**	
C	-1.61121200	0.02454200	0.00015900	-980.08	75.98	139.39
C	-1.16086500	1.30619700	-0.00010700	166.1	187.41	243.06
C	0.27422300	1.24022500	-0.00059600	306.68	338.13	443.41
C	0.60353900	-0.07907800	-0.00039400	603.57	632.29	665.52
O	-0.54174400	-0.83410000	-0.00026000	707.93	812.52	859.23
H	-1.77276200	2.19474200	-0.00002400	896.13	968.03	985.96
C	1.88976500	-0.83585200	-0.00086300	1003.68	1019.34	1065.34
C	3.12531300	0.06744300	0.00115900	1075.95	1117.33	1137.81
H	3.14644800	0.71117300	-0.88196200	1192.47	1222.9	1289.35

H	3.14538600	0.70865500	0.88613400
H	4.03805600	-0.53227700	0.00081500
H	1.91177100	-1.49916400	0.87249900
H	1.91283200	-1.49650500	-0.87624100
C	-2.96306400	-0.59384000	0.00058200
H	-3.72770500	0.18429800	0.00156500
H	-3.11435500	-1.22278300	-0.88260300
H	-3.11336700	-1.22407900	0.88301500
H	0.66109571	0.44581012	1.01384211

1337.88	1382.2	1398.43
1417.24	1423.39	1475.15
1484.52	1492.97	1494.59
1509.87	1601.34	2156.97
3028.98	3033.18	3036.21
3059.9	3084.43	3096.18
3122.28	3126.47	3212.77

TS21

C	1.53840800	-0.04334000	-0.00122400
C	1.06268100	1.29080000	-0.14884800
C	-0.27922300	1.38064500	0.14841200
C	-0.72471600	0.07432500	0.50191500
O	0.67148300	-0.90659100	0.36206600
H	1.72379900	2.09338500	-0.45753900
C	2.94181600	-0.50890100	-0.22299100
H	3.57865800	0.30474300	-0.57434700
H	3.35151400	-0.90715400	0.71108800
H	2.95585900	-1.32379000	-0.95323200
H	-0.90778700	-0.05535500	1.57350700
C	-1.75998500	-0.63196500	-0.35877700
H	-1.75059500	-1.69754600	-0.10994300
H	-1.46686200	-0.53596200	-1.40975700
C	-3.16269800	-0.04790100	-0.14988400
H	-3.48322200	-0.14806600	0.89266000
H	-3.18030800	1.01397600	-0.40513200
H	-3.89062400	-0.57347800	-0.77546000

Frequencies

-290.1	86.55	119.01
129.96	202.31	264.7
279.66	386.94	402.34
539.26	651.1	659.33
750.82	812.47	862.16
902.64	968.46	993.55
1024.3	1038.36	1061.95
1089.61	1121.67	1154.37
1225.84	1268.3	1315.86
1359.34	1361.61	1404.15
1410.59	1422.05	1476.77
1489.59	1494.52	1496.64
1513.85	1555.14	3018.75
3027.26	3028.7	3059.87
3071.01	3081.59	3085.54
3105.75	3116.85	3165.89

TS22

C	-1.61121200	0.02454200	0.00015900
C	-1.16086500	1.30619700	-0.00010700
C	0.27422300	1.24022500	-0.00059600
C	0.60353900	-0.07907800	-0.00039400
O	-0.54174400	-0.83410000	-0.00026000
H	0.96145300	2.07091600	-0.00075500
C	1.88976500	-0.83585200	-0.00086300
C	3.12531300	0.06744300	0.00115900
H	3.14644800	0.71117300	-0.88196200
H	3.14538600	0.70865500	0.88613400
H	4.03805600	-0.53227700	0.00081500
H	1.91177100	-1.49916400	0.87249900
H	1.91283200	-1.49650500	-0.87624100

Frequencies

-971.08	58.1	162.88
179.06	201.69	244.16
305.25	343.49	454.66
608.51	627.01	649.19
699.75	799.68	862.67
885.55	943.71	973.4
1007.46	1036.19	1082.98
1099.65	1112.24	1146.46
1197	1212.92	1284.71
1309.58	1387.15	1399.22
1420.2	1428.38	1475.95
1481.07	1498.29	1500.66
1508.78	1589.22	2171.26

C	-2.96306400	-0.59384000	0.00058200
H	-3.72770500	0.18429800	0.00156500
H	-3.11435500	-1.22278300	-0.88260300
H	-3.11336700	-1.22407900	0.88301500
H	-1.60654499	0.67733286	1.00072264

3019.16	3037.54	3041.33
3044.22	3102.9	3103.05
3105.32	3138.16	3223.57

TS23

C	-1.50135279	-0.15617210	0.00012224
C	-1.12725655	1.32584805	-0.00012356
C	0.22991007	1.26345815	-0.00058600
C	0.58785622	-0.17054273	-0.00036644
O	-0.27831289	-1.10697509	-0.00034621
H	0.91714007	2.09414915	-0.00074500
C	1.87408222	-0.92731673	-0.00083544
C	3.10963022	-0.02402173	0.00118656
H	3.13076522	0.61970827	-0.88193444
H	3.12970322	0.61719027	0.88616156
H	4.02237322	-0.62374173	0.00084256
H	1.89608822	-1.59062873	0.87252656
H	1.89714922	-1.58796973	-0.87621344
C	-2.85320479	-0.77455410	0.00054524
H	-3.61784579	0.00358390	0.00152824
H	-3.00449579	-1.40349710	-0.88263976
H	-3.00350779	-1.40479310	0.88297824
H	-1.55282069	-0.12404444	1.06840070

Frequencies

-209.32	36.73	122.16
203.96	215.67	235.7
267.69	340.41	482.29
534.41	619.59	628.91
742.46	804	875.15
901.45	945.33	1007.61
1010.19	1045.66	1082.5
1105.26	1129.77	1146.41
1224.07	1285.85	1297.27
1347.36	1375.29	1391.72
1405.89	1424.82	1460
1490.34	1491.39	1503.49
1507.55	1528.03	3027.12
3039.49	3045.98	3051.41
3078.05	3105.68	3106.29
3118.71	3124.23	3191.65

5ef2y

C	2.14360200	-0.60639000	0.00034000
C	2.17467900	0.75687700	0.00016600
C	0.81339700	1.17319600	-0.00018100
C	0.05187000	0.03230200	-0.00008500
O	0.87346400	-1.06145400	0.00026600
H	3.05437400	1.38014000	0.00023100
H	0.44440100	2.18639100	-0.00041800
C	1.40030000	-0.21132300	-0.00021700
C	2.28795200	1.02188600	0.00023400
H	2.09865000	1.63893000	0.88325900
H	2.09869100	1.63954300	-0.88237100
H	3.32773500	0.69902100	0.00015000
H	1.63904524	-0.79128425	-0.86713433
H	1.63909094	-0.79195106	0.86624128

Frequencies

46.26	192.71	200.43
274.33	425.83	476.34
620.17	644.74	773.34
796.73	839.7	879.03
915.11	992.14	1012.57
1071.6	1113.24	1117.05
1148.67	1221.75	1280.26
1315.85	1387.16	1413.66
1477.73	1491.37	1500.37
1511.76	1633.46	3012.98
3033.45	3034.34	3098.21
3103.74	3241.93	3273.59

5mf2ym

C	2.14360200	-0.60639000	0.00034000
C	2.17467900	0.75687700	0.00016600

Frequencies

135.46	181.29	272.17
283.9	409.95	535.61

C	0.81339700	1.17319600	-0.00018100
C	0.05187000	0.03230200	-0.00008500
O	0.87346400	-1.06145400	0.00026600
H	3.05437400	1.38014000	0.00023100
H	0.44440100	2.18639100	-0.00041800
C	1.40030000	-0.21132300	-0.00021700
H	1.63904524	-0.79128425	-0.86713433
H	1.63909094	-0.79195106	0.86624128
C	3.24340335	-1.68436814	0.00068590
H	4.10966282	-1.30994394	0.50496862
H	2.88657569	-2.55779183	0.50536903
H	3.49811925	-1.93435344	-1.00803961

610.65	624.03	653.03
705.8	725.7	751.12
856.13	910.76	956.75
979.67	1022.39	1040.01
1052.84	1221.98	1260.09
1310.76	1399.33	1409.79
1424.94	1469.74	1486.64
1525.78	1577.99	3018.03
3061.13	3119.33	3171.14
3234.93	3251.22	3271.3

5mef2y

C	2.14360200	-0.60639000	0.00034000
C	2.17467900	0.75687700	0.00016600
C	0.81339700	1.17319600	-0.00018100
C	0.05187000	0.03230200	-0.00008500
O	0.87346400	-1.06145400	0.00026600
H	3.05437400	1.38014000	0.00023100
H	0.44440100	2.18639100	-0.00041800
C	3.24340335	-1.68436814	0.00068590
H	4.10966282	-1.30994394	0.50496862
H	2.88657569	-2.55779183	0.50536903
H	3.49811925	-1.93435344	-1.00803961

Frequencies

134.12	231.01	335.9
476.1	622.37	650.49
774.03	836.05	880.74
912.29	988.26	1021.9
1064.86	1115.5	1185.14
1224.29	1343.43	1418.57
1470.13	1475.08	1497.57
1641.89	3024.59	3072.36
3114.45	3235.35	3273.61

5ef2ym

C	2.14360200	-0.60639000	0.00034000
C	2.17467900	0.75687700	0.00016600
C	0.81339700	1.17319600	-0.00018100
C	0.05187000	0.03230200	-0.00008500
O	0.87346400	-1.06145400	0.00026600
H	3.05437400	1.38014000	0.00023100
H	0.44440100	2.18639100	-0.00041800
C	-1.40030000	-0.21132300	-0.00021700
C	-2.28795200	1.02188600	0.00023400
H	-2.09865000	1.63893000	0.88325900
H	-2.09869100	1.63954300	-0.88237100
H	-3.32773500	0.69902100	0.00015000
H	-1.63904524	-0.79128425	-0.86713433
H	-1.63909094	-0.79195106	0.86624128
C	3.24340335	-1.68436814	0.00068590
H	4.10966282	-1.30994394	0.50496862
H	2.88657569	-2.55779183	0.50536903

Frequencies

57.08	160.75	182.15
239.02	290.03	324.05
453.2	535.71	607.73
633.43	652.53	704.99
719.35	749.3	795.62
860.25	928.39	945.28
961.61	1011.13	1042.38
1082.24	1106.34	1212.77
1228.6	1279.58	1308.14
1365.17	1403.22	1419.6
1423.54	1481.44	1500.99
1509.42	1524.67	1569.9
3005.2	3022.77	3034.33
3097.02	3103.72	3170.65
3239.65	3253.46	3270.72

	25mf2ye				Frequencies	
C	-1.55049200	0.01382700	-0.01716300	56.32	129.29	166.7
C	-1.12546400	1.30339400	0.04236300	167.86	210.33	271.43
C	0.30791900	1.26397200	0.11520500	318.01	449.71	493.86
C	0.66114200	-0.04845800	0.09412800	610.46	635.19	658.62
O	-0.46616300	-0.82490000	0.01224200	690.67	801.95	840.33
H	-1.75413800	2.18009500	0.03390600	862.43	962.17	975.33
H	0.98320700	2.10177300	0.18351300	1003.42	1034.85	1044.4
C	1.96380300	-0.77952600	0.16650800	1065.04	1098.41	1165.41
C	3.13968300	0.03795700	-0.24470000	1223.49	1233.6	1240.93
H	3.05782400	0.74239800	-1.06287400	1345.93	1397.01	1419.49
H	4.12183800	-0.17286800	0.15751700	1458.2	1459.97	1473.76
H	2.11662300	-1.17427300	1.18030600	1494.55	1597.01	1653.18
H	1.87464000	-1.67542900	-0.47294600	2925.43	2995.99	3021.18
C	-2.88832300	-0.62785000	-0.10923400	3066.09	3115.69	3143.14
H	-3.66654000	0.13652900	-0.12285200	3236.88	3249.22	3251.89
H	-2.98115800	-1.22860400	-1.01969400			
H	-3.07259400	-1.29032100	0.74254700			

	2e5mf3y				Frequencies	
C	2.14360200	-0.60639000	0.00034000	49.94	127.55	161.53
C	2.17467900	0.75687700	0.00016600	166.99	235.01	277.44
C	0.81339700	1.17319600	-0.00018100	303.5	448.24	567.54
C	0.05187000	0.03230200	-0.00008500	628.59	632.54	688.87
O	0.87346400	-1.06145400	0.00026600	788.02	803.02	939.53
H	3.05437400	1.38014000	0.00023100	958.57	995.23	1027.54
C	-1.40030000	-0.21132300	-0.00021700	1059.03	1066.21	1114.24
C	-2.28795200	1.02188600	0.00023400	1123.64	1165.62	1220.07
H	-2.09865000	1.63893000	0.88325900	1280.33	1326.49	1380.6
H	-2.09869100	1.63954300	-0.88237100	1416.09	1419.17	1473.24
H	-3.32773500	0.69902100	0.00015000	1488.64	1490.1	1498.94
H	-1.63904524	-0.79128425	-0.86713433	1508.97	1594.25	1625.59
H	-1.63909094	-0.79195106	0.86624128	3010.23	3022.67	3029.62
C	3.24340335	-1.68436814	0.00068590	3035.43	3069.35	3099.61
H	4.10966282	-1.30994394	0.50496862	3103.29	3117.01	3252.15
H	2.88657569	-2.55779183	0.50536903			
H	3.49811925	-1.93435344	-1.00803961			

	5e2mf3y				Frequencies	
C	2.14360200	-0.60639000	0.00034000	54.37	128.89	169.86
C	2.17467900	0.75687700	0.00016600	175.84	229.79	281.19
C	0.81339700	1.17319600	-0.00018100	303.55	449.24	570.18
C	0.05187000	0.03230200	-0.00008500	620.29	630.4	685.71
O	0.87346400	-1.06145400	0.00026600	775.58	813.74	939.09

H	0.44440100	2.18639100	-0.00041800	952.04	987.47	1023.91
C	-1.40030000	-0.21132300	-0.00021700	1063.47	1081.91	1109.23
C	-2.28795200	1.02188600	0.00023400	1128.31	1187.11	1198.55
H	-2.09865000	1.63893000	0.88325900	1281.45	1314.44	1383.83
H	-2.09869100	1.63954300	-0.88237100	1416.07	1418.64	1474.24
H	-3.32773500	0.69902100	0.00015000	1485.89	1489.71	1500.19
H	-1.63904524	-0.79128425	-0.86713433	1510.2	1585.12	1635.47
H	-1.63909094	-0.79195106	0.86624128	3010	3023.32	3030.31
C	3.24340335	-1.68436814	0.00068590	3033.94	3069.13	3097.18
H	4.10966282	-1.30994394	0.50496862	3102.84	3121.49	3258.82
H	2.88657569	-2.55779183	0.50536903			
H	3.49811925	-1.93435344	-1.00803961			

**2-ethyl-5-methyl-2,3-dihydrofuran-2-yl**					**Frequencies**	
C	-1.61121200	0.02454200	0.00015900	63.35	108.97	164.08
C	-1.16086500	1.30619700	-0.00010700	183.67	233.13	248.94
C	0.27422300	1.24022500	-0.00059600	296.37	336.78	436.22
C	0.60353900	-0.07907800	-0.00039400	571.82	611.53	652.1
O	-0.54174400	-0.83410000	-0.00026000	753.23	782.52	835.71
H	-1.77276200	2.19474200	-0.00002400	934.82	939.49	961.26
H	0.66310029	1.71503375	0.87589194	986.7	1025.88	1061.29
C	1.88976500	-0.83585200	-0.00086300	1068.5	1074.66	1129.55
C	3.12531300	0.06744300	0.00115900	1167.12	1207.14	1231.73
H	3.14644800	0.71117300	-0.88196200	1276.53	1312.99	1337.33
H	3.14538600	0.70865500	0.88613400	1382.02	1413.5	1420.62
H	4.03805600	-0.53227700	0.00081500	1467.67	1471.9	1478.56
H	1.91177100	-1.49916400	0.87249900	1491.05	1499.3	1508.45
H	1.91283200	-1.49650500	-0.87624100	1718.26	2879	2903.15
C	-2.96306400	-0.59384000	0.00058200	2974.54	3028.08	3029.21
H	-3.72770500	0.18429800	0.00156500	3048.96	3077.82	3090.47
H	-3.11435500	-1.22278300	-0.88260300	3096.31	3123.29	3232.09
H	-3.11336700	-1.22407900	0.88301500			
H	0.66248338	1.71461128	-0.87758604			

**(Z)-5-oxohept-2-en-2-yl**					**Frequencies**	
C	-1.61303355	1.35173280	1.30101773	22.14	59.8	65.17
C	-1.22288364	1.33356357	-0.00009908	123.26	187.78	208.53
C	0.28012895	1.26446903	-0.00061123	226.45	248.45	282.22
C	0.69123328	1.24532268	-1.37866997	345.09	477.77	551.05
O	-0.38133008	1.29492559	-2.15897292	581.21	740.79	796.46
H	-1.87455278	1.36331561	-0.85938501	824.29	900.72	930.94
H	0.60601642	0.37191117	0.49136218	961.92	1020.57	1048.54
C	2.00922931	1.18480728	-2.07602839	1051.55	1077.12	1100.76
C	3.20094457	1.12751848	-1.11735351	1171.36	1193.63	1264.58

H	3.23357669	2.01002498	-0.47326104	1285.68	1329.92	1359.76
H	3.15083549	0.24386600	-0.47596136	1397.35	1408.36	1440.04
H	4.13908779	1.08445881	-1.67484535	1455.51	1475.39	1484.05
H	2.02099038	0.31001566	-2.73771530	1500.85	1508.65	1771.36
H	2.10282871	2.05683970	-2.73487566	1792.37	2963.6	2975.3
C	-2.93343485	1.41245817	1.98127733	3032.07	3035.56	3036.33
H	-3.73238206	1.44984046	1.23934640	3045.91	3063.26	3069.29
H	-3.09559594	0.53580268	2.61661420	3082.86	3100.28	3110.2
H	-3.01406947	2.29953786	2.61772676			
H	0.68653989	2.12353940	0.49104744			

2-ethyl-5-methyl-2,3-dihydrofuran-3-yl					Frequencies	
C	-1.61121200	0.02454200	0.00015900	78.78	113.43	144.39
C	-1.16086500	1.30619700	-0.00010700	196.77	242.71	252.56
C	0.27422300	1.24022500	-0.00059600	305.4	415.15	458.6
C	0.60353900	-0.07907800	-0.00039400	549.31	575.63	646.92
O	-0.54174400	-0.83410000	-0.00026000	746.97	811.09	860.7
H	-1.77276200	2.19474200	-0.00002400	871.47	928.97	946.28
H	0.96145300	2.07091600	-0.00075500	965.13	1020.37	1040.47
C	1.88976500	-0.83585200	-0.00086300	1049.3	1058.76	1103.17
C	3.12531300	0.06744300	0.00115900	1135.21	1248.25	1272.97
H	3.14644800	0.71117300	-0.88196200	1280.55	1313.97	1360.33
H	3.14538600	0.70865500	0.88613400	1361.54	1412.79	1418.12
H	4.03805600	-0.53227700	0.00081500	1422.69	1464.05	1472.96
H	1.91177100	-1.49916400	0.87249900	1490.62	1503.41	1509.58
H	1.91283200	-1.49650500	-0.87624100	1539.24	2937.23	3003.07
C	-2.96306400	-0.59384000	0.00058200	3024.47	3030.31	3038.68
H	-3.72770500	0.18429800	0.00156500	3064.19	3089.01	3095.14
H	-3.11435500	-1.22278300	-0.88260300	3113.39	3217.39	3242.4
H	-3.11336700	-1.22407900	0.88301500			
H	0.66031319	-0.04965497	1.06769353			

2-methylfuran					Frequencies	
C	-1.61121200	0.02454200	0.00015900	123.11	241.32	337.29
C	-1.16086500	1.30619700	-0.00010700	615.44	641.08	659.1
C	0.27422300	1.24022500	-0.00059600	736.81	811.35	871.32
C	0.60353900	-0.07907800	-0.00039400	905.46	938.26	991.56
O	-0.54174400	-0.83410000	-0.00026000	1035.18	1065.73	1109.95
H	-1.77276200	2.19474200	-0.00002400	1169.32	1235.82	1253.34
H	0.96145300	2.07091600	-0.00075500	1407.96	1421	1474.28
C	-2.96306400	-0.59384000	0.00058200	1492.22	1542.68	1639.37
H	-3.72770500	0.18429800	0.00156500	3024.54	3070.66	3117.89
H	-3.11435500	-1.22278300	-0.88260300	3237.43	3249.04	3278.27
H	-3.11336700	-1.22407900	0.88301500			

H	1.60048277	-0.46766920	-0.00034692

(E)-2-oxohept-4-en-3-yl

C	-1.55101491	1.32436623	1.30100981
C	-1.16086500	1.30619700	-0.00010700
C	0.27422300	1.24022500	-0.00059600
C	0.66291207	1.22212260	-1.30351679
O	-0.44413721	1.27350292	2.10925652
H	-1.81253414	1.33594905	-0.85939293
H	0.92218380	1.21030934	0.86055700
C	1.98090810	1.16160720	-2.00087522
C	3.17262337	1.10431839	-1.04220034
H	3.20525548	1.98682490	-0.39810786
H	3.12251428	0.22066592	-0.40080819
H	4.11076659	1.06125872	-1.59969217
H	1.99266917	0.28681558	-2.66256212
H	2.07450751	2.03363962	-2.65972248
C	-2.87141622	1.38509161	1.98126941
H	-3.67036342	1.42247389	1.23933848
H	-3.03357731	0.50843611	2.61660628
H	-2.95205083	2.27217129	2.61771884
H	-0.16323598	1.25979471	-1.98245729

Frequencies

56.91	83.7	98.27
101.31	208.8	223.95
232.93	292.9	345.82
398.96	568.92	574.91
620.85	727.83	786.92
854.21	861.77	934.86
958.68	1020.55	1030.26
1034.89	1086.37	1101.9
1190.4	1214.9	1281.84
1282.8	1308.03	1377.56
1382.61	1417.18	1450.36
1463.98	1473.92	1478.29
1502.87	1509.63	1547.03
1680.24	2982.33	2992.3
3025.57	3033.68	3080.52
3096.96	3099.32	3121.21
3139.42	3150.07	3168.29

5-ethyl-2-methyl-2,3-dihydrofuran-2-yl

C	-1.61710058	0.00778354	0.00016248
C	-1.15497642	1.32295546	-0.00011048
C	0.27422300	1.24022500	-0.00059600
C	0.60353900	-0.07907800	-0.00039400
O	-0.54174400	-0.83410000	-0.00026000
H	-1.49475141	1.83260366	-0.87744261
H	0.96145300	2.07091600	-0.00075500
C	1.88976500	-0.83585200	-0.00086300
C	3.12531300	0.06744300	0.00115900
H	3.14644800	0.71117300	-0.88196200
H	3.14538600	0.70865500	0.88613400
H	4.03805600	-0.53227700	0.00081500
H	1.91177100	-1.49916400	0.87249900
H	1.91283200	-1.49650500	-0.87624100
C	-2.96895258	-0.61059846	0.00058548
H	-3.73359358	0.16753954	0.00156848
H	-3.12024358	-1.23954146	-0.88259952
H	-3.11925558	-1.24083746	0.88301848
H	-1.49414650	1.83275531	0.87736760

Frequencies

60.72	128.7	156.44
177.32	194.12	261.02
294.8	329.92	440.62
569.44	606.62	646.47
749.28	806.46	848.76
925.47	929.91	950.95
986.38	1018.41	1032.29
1085.63	1113.85	1128.87
1168.07	1205.27	1234.8
1282.61	1289.63	1344.41
1397.14	1413.4	1417.14
1465.36	1473.47	1482.92
1485.53	1499.6	1510.52
1709.59	2880.56	2926.7
2968.32	3016.11	3033.86
3036.48	3054.18	3097.62
3098.84	3101.21	3239.67

(E)-6-oxohept-4-en-3-yl

Frequencies

C	-1.53921387	1.34544405	1.33969989	36.37	52.71	70.57
C	-1.15497642	1.32295546	-0.00011048	117.23	137.09	191.53
C	0.27422300	1.24022500	-0.00059600	231.49	297.29	309.02
C	0.67809979	1.21660724	-1.29880061	381.86	470.43	563.89
O	-0.41861350	1.28059567	2.11765192	594.28	712.34	795.19
H	-1.57359780	0.46822374	-0.48907859	814.88	844.56	897.31
H	0.91152024	1.20320943	0.86820473	942.83	1002.8	1041.67
C	2.00346406	1.13995903	-1.98043029	1048.35	1097.59	1103.55
C	3.18295676	1.06918072	-1.00764449	1169.64	1203.95	1269.49
H	3.21843201	1.95168189	-0.36369504	1279.37	1332.56	1352.51
H	3.11474256	0.18662890	-0.36640693	1384.02	1401.62	1444.5
H	4.12706990	1.01459560	-1.55393637	1465.27	1473.92	1477.41
H	2.01267618	0.26463274	-2.64145016	1494.73	1506.57	1763.69
H	2.11523143	2.01036298	-2.63859907	1798.12	2977.03	3007.49
C	-2.85063791	1.42138842	2.03561637	3027.52	3028.7	3032.19
H	-3.65782872	1.46876645	1.30324086	3044.29	3066.82	3085.45
H	-3.01566160	0.54628811	2.67235858	3099.31	3106.22	3138.51
H	-2.91316600	2.30892903	2.67345812			
H	-1.47218894	2.22010142	-0.48940115			

5-ethyl-2-methyl-2,3-dihydrofuran-3-yl **Frequencies**

C	-1.61121200	0.02454200	0.00015900	53.24	108.88	179.95
C	-1.16086500	1.30619700	-0.00010700	193.72	234.5	260.18
C	0.27422300	1.24022500	-0.00059600	316.01	420.03	494.18
C	0.60353900	-0.07907800	-0.00039400	545.1	567.42	629.38
O	-0.54174400	-0.83410000	-0.00026000	750.12	791.29	840.84
H	-1.77276200	2.19474200	-0.00002400	871.99	907.99	919.45
H	0.96145300	2.07091600	-0.00075500	991.66	1022.92	1057.36
C	1.88976500	-0.83585200	-0.00086300	1077.36	1087.73	1098.22
C	3.12531300	0.06744300	0.00115900	1119.67	1217.12	1267.37
H	3.14644800	0.71117300	-0.88196200	1273.33	1328.53	1346.51
H	3.14538600	0.70865500	0.88613400	1384.03	1399.59	1415.28
H	4.03805600	-0.53227700	0.00081500	1424.45	1475.19	1484.33
H	1.91177100	-1.49916400	0.87249900	1494.42	1500.98	1509.73
H	1.91283200	-1.49650500	-0.87624100	1524.48	2947.77	2990.68
C	-2.96306400	-0.59384000	0.00058200	3003.36	3029.74	3032.45
H	-3.72770500	0.18429800	0.00156500	3094.11	3099.18	3100.84
H	-3.11435500	-1.22278300	-0.88260300	3109.67	3223.77	3240.02
H	-3.11336700	-1.22407900	0.88301500			
H	-1.66267991	0.05666966	1.06843746			

(Z)-5-oxohept-2-en-4-yl **Frequencies**

C	-1.55101491	1.32436623	1.30100981	47.48	84.55	107.76
C	-1.16086500	1.30619700	-0.00010700	130.32	185.33	211.97

C	0.27422300	1.24022500	-0.00059600	246.39	264.31	335.79
C	0.66291207	1.22212260	-1.30351679	346.72	521.39	549.45
O	-0.44558021	1.27338711	-2.10995848	658.96	761.71	793.09
H	-1.81253414	1.33594905	-0.85939293	837.68	871.03	938.53
H	0.92218380	1.21030934	0.86055700	1007.1	1015.86	1040.41
C	1.98090810	1.16160720	-2.00087522	1047.44	1081.5	1121.65
C	3.17262337	1.10431839	-1.04220034	1141.3	1213.64	1277.51
H	3.20525548	1.98682490	-0.39810786	1284.15	1301.43	1362.27
H	3.12251428	0.22066592	-0.40080819	1408.71	1415.9	1448.38
H	4.11076659	1.06125872	-1.59969217	1460.85	1474.18	1490.83
H	1.99266917	0.28681558	-2.66256212	1491.17	1501.45	1546.09
H	2.07450751	2.03363962	-2.65972248	1683.4	3002.13	3006.05
C	-2.87141622	1.38509161	1.98126941	3029.71	3038.1	3040.45
H	-3.67036342	1.42247389	1.23933848	3099.71	3104.58	3111.16
H	-3.03357731	0.50843611	2.61660628	3128.04	3145.22	3153.66
H	-2.95205083	2.27217129	2.61771884			
H	-1.55516471	2.39394327	1.27121508			

**2-ethyl-5-methylfuran-3(2H)carbene**					**Frequencies**	
C	-1.51697900	-0.00168100	-0.00551700	17.65	68.71	110.73
C	-1.05451700	1.30792700	-0.15116600	179.61	237.88	270.13
C	0.30828700	1.38602900	0.14487100	349.89	450.76	536.93
C	0.64646700	-0.00155400	0.49940300	635.19	674.83	767.78
O	-0.59850600	-0.83871000	0.37285900	826.54	874.23	903.74
H	-1.69936700	2.11727000	-0.46978200	946.9	985.01	1015.13
C	1.71275900	-0.65879200	-0.37159900	1025.81	1058.68	1079.82
C	3.08497400	-0.03245500	-0.14324100	1132.94	1192.95	1202.97
H	3.05005000	1.03780200	-0.34471100	1228.56	1257.91	1318.47
H	3.40843000	-0.17153600	0.89132400	1340.58	1370.19	1408.01
H	3.82966400	-0.49428200	-0.79348300	1423.87	1461.03	1471.92
H	1.73083100	-1.72765700	-0.14576500	1492.15	1496.37	1509.03
H	1.41082000	-0.54677100	-1.41714300	1513.3	2811.44	3037.65
C	-2.88073400	-0.55272800	-0.21811000	3044.71	3048	3077.53
H	-3.55929800	0.21971000	-0.57428300	3102.98	3109.78	3128.62
H	-2.83724200	-1.36994300	-0.94056800	3148.35	3178.48	0
H	-3.25514700	-0.96347100	0.72220900			
H	0.90776000	-0.07192300	1.56148400			

**hepta-3,4-dien-2-one**					**Frequencies**	
C	-1.06351649	-0.52420419	-2.09109098	44.73	63.91	86.84
C	-0.78945100	-0.61581700	-0.62164700	120.01	158.08	203.27
C	0.41583600	-0.83157700	-0.14907000	280.63	341.1	372.73
C	1.60979700	-1.04530900	0.31906600	435.16	565.44	603.43
O	-0.20608931	-0.70314816	-2.92865505	667.56	719.7	812.52

H	-1.62710536	-0.49213856	0.06135216	845.44	867.13	907.77
C	1.96633640	-1.99381907	1.44398160	957.04	1012.03	1039.44
C	0.78763519	-2.76699675	2.03231251	1085.84	1105.12	1144.76
H	0.04351209	-2.08970500	2.45974811	1178.07	1290.14	1297.82
H	0.29143438	-3.36601547	1.26523213	1363.81	1380.19	1416.46
H	1.12676190	-3.43830557	2.82479062	1442.96	1467.45	1475.47
H	2.72001804	-2.69188628	1.06101617	1482.61	1498.91	1507.51
H	2.47025853	-1.41944467	2.23166769	1767.66	2034.44	3009.04
C	-2.49147210	-0.17268671	-2.46982802	3024.89	3030.74	3033.25
H	-3.21604060	-0.72794956	-1.86551821	3080.33	3097.01	3102.21
H	-2.66198561	0.89550576	-2.28572193	3111.37	3126.79	3142.76
H	-2.64703736	-0.37857944	-3.52911333			
H	2.43899164	-0.54411995	-0.17869623			

5-ethyl-2-methylfuran-3(2H)carbene				Frequencies		
C	-1.61121200	0.02454200	0.00015900	40.42	122.36	166.17
C	-1.16086500	1.30619700	-0.00010700	208.34	230.28	264.21
C	0.27422300	1.24022500	-0.00059600	276.87	353.76	495.96
C	0.60353900	-0.07907800	-0.00039400	535.38	622.9	628.36
O	-0.54174400	-0.83410000	-0.00026000	753.36	803.81	877.67
H	0.96145300	2.07091600	-0.00075500	899.82	949.07	1004.57
C	1.88976500	-0.83585200	-0.00086300	1014.56	1044.26	1083.09
C	3.12531300	0.06744300	0.00115900	1105.67	1129.7	1167.07
H	3.14644800	0.71117300	-0.88196200	1221.02	1284.79	1293.6
H	3.14538600	0.70865500	0.88613400	1333.39	1364.83	1403.38
H	4.03805600	-0.53227700	0.00081500	1405.49	1427.23	1462.95
H	1.91177100	-1.49916400	0.87249900	1489.78	1492.01	1503.4
H	1.91283200	-1.49650500	-0.87624100	1504.8	1515.57	3026.02
C	-2.96306400	-0.59384000	0.00058200	3039.89	3045.77	3050.16
H	-3.72770500	0.18429800	0.00156500	3068.37	3106.46	3106.59
H	-3.11435500	-1.22278300	-0.88260300	3117.75	3126.42	3191.74
H	-3.11336700	-1.22407900	0.88301500			
H	-1.66267991	0.05666966	1.06843746			

hepta-4,5-dien-3-one				Frequencies		
C	-1.55101491	1.32436623	1.30100981	43.19	47.8	91.77
C	-1.16086500	1.30619700	-0.00010700	149.63	180.03	206.85
C	0.27422300	1.24022500	-0.00059600	221.64	283.04	374.03
C	0.66291207	1.22212260	-1.30351679	434.85	566.62	610.53
O	-0.44558021	1.27338711	-2.10995848	673.11	778.08	807.27
H	0.92218380	1.21030934	0.86055700	868.67	885.81	904.95
C	1.98090810	1.16160720	-2.00087522	958.98	1025.13	1057.4
C	3.17262337	1.10431839	-1.04220034	1076.64	1087.45	1146.57
H	3.20525548	1.98682490	-0.39810786	1182.19	1277.27	1311.69

H	3.12251428	0.22066592	-0.40080819	1332.82	1406.98	1410.8
H	4.11076659	1.06125872	-1.59969217	1436.61	1481.15	1484.89
H	1.99266917	0.28681558	-2.66256212	1498.79	1501.19	1507.8
H	2.07450751	2.03363962	-2.65972248	1762.25	2038.13	3024.82
C	-2.87141622	1.38509161	1.98126941	3027.25	3030.99	3075.3
H	-3.67036342	1.42247389	1.23933848	3085.4	3099.83	3107.18
H	-3.03357731	0.50843611	2.61660628	3108.85	3125.19	3128.19
H	-2.95205083	2.27217129	2.61771884			
H	-1.55516471	2.39394327	1.27121508			
		**C_2H_5**			**Frequencies**	
C	1.88976500	-0.83585200	-0.00086300	108.56	477.33	813.2
C	3.12531300	0.06744300	0.00115900	979.78	1061.86	1191.62
H	3.14644800	0.71117300	-0.88196200	1400.78	1464.92	1482.59
H	3.14538600	0.70865500	0.88613400	1482.82	2943.59	3033.76
H	4.03805600	-0.53227700	0.00081500	3076.9	3138.97	3238.86
H	1.91177100	-1.49916400	0.87249900			
H	1.91283200	-1.49650500	-0.87624100			
		**CH_3**			**Frequencies**	
C	3.12531300	0.06744300	0.00115900	505.58	1403.12	1403.38
H	3.14644800	0.71117300	-0.88196200	3103.78	3282.69	3283.02
H	3.14538600	0.70865500	0.88613400			
H	4.03805600	-0.53227700	0.00081500			
		**CH_4**			**Frequencies**	
C	1.53049956	-0.12565761	-1.51916421	1341.21	1341.21	1341.21
H	1.88715398	-1.13446761	-1.51916421	1561.07	1561.07	3028.46
H	1.88717240	0.37874058	-0.64551271	3134.84	3134.84	3134.84
H	1.88717240	0.37874058	-2.39281572			
H	0.46049956	-0.12564443	-1.51916421			
		**H**			**Frequencies**	
H	1.88717240	0.37874058	-0.64551271			
		**H_2**			**Frequencies**	
H	2.48717240	0.37874058	-0.64551271	4419.26		
H	1.88717240	0.37874058	-0.64551271			
